# The incidence of vestibular neuritis in Italy

**DOI:** 10.3389/fneur.2023.1177621

**Published:** 2023-05-18

**Authors:** Marco Mandalà, Lorenzo Salerni, Fabio Ferretti, Ilaria Bindi, Giacomo Gualtieri, Giulia Corallo, Francesca Viberti, Roberto Gusinu, Claudio Fantino, Silvia Ponzo, Serena Astore, Simone Boccuzzi, Daniele Nuti

**Affiliations:** ^1^Department of Medicine, Surgery and Neuroscience, University of Siena, Siena, Italy; ^2^University Hospital Le Scotte, Siena, Italy; ^3^Department of Otorhinolaryngology, ENT Clinic, Hospital Santa Croce e Carle di Cuneo, Cuneo, Italy; ^4^Department of Otorhinolaryngology, ENT Clinic, Hospital Misericordia di Grosseto, Grosseto, Italy

**Keywords:** acute vestibular neuronitis, dizziness, prospective study, epidemiology, incidence

## Abstract

**Objective:**

This study aims to estimate the incidence of Vestibular neuritis (VN) in three different districts in Italy, its epidemiological features, and the prevalence of comorbidities associated with it.

**Methods:**

An observational prospective study of 198 patients referred to ENT departments in Siena, Grosseto, and Cuneo was carried out over a 2-year period. Each patient underwent a complete otoneurologic examination in the first 48 h from the onset of symptoms and a brain MRI in the early stages of the disease. The follow-up lasted for 1 year.

**Results:**

The total VN incidence rate of the three municipalities was 48.497 (95% CI: 48.395–48.598) and its standardized value was 53.564 (95% CI: 53.463–53.666). The total VN incidence rate for the whole sample (municipality and district of the three centers) was 18.218 (95% CI: 18.164–18.272), and its standardized value was 20.185 (95% CI: 20.129–20.241). A significant difference was highlighted between patients living in the city compared to those living in the surrounding area (*p* < 0.000), this may be due to the ease of reaching the otoneurological referral center.

**Conclusion:**

The total incidence rate for the three municipalities was 48.497. This result is higher than previously reported studies.

## 1. Introduction

Vestibular neuritis (VN), also known as acute unilateral peripheral vestibulopathy or unilateral sudden vestibular loss, is one of the most impairing acute vestibular disorders and a common cause of peripheral vertigo in adults. The etiology is thought to be viral, though in rare cases, the cause may be labyrinthine ischemia (1). Sudden and severe vestibular symptoms, often debilitating, require clinical attention to differentiate them from acute vascular lesions such as cerebellar infarction (1). Although it is considered one of the most frequent and invalidating causes of vertigo in specialized dizziness units (2, 3), the scarcity of epidemiological data is surprising as reported by a recent review by Neuhauser et al., possibly due to the lack of standardized interviews/questionnaires for its diagnosis (2). The most reliable data come from a prospective, population-based study performed in Croatia in the years 2011 and 2012. According to that study, the annual incidence of VN ranged from 11.7 to 15.5 per 100.000 inhabitants per year with an uneven distribution throughout the different seasons of the year (4); previously Sekitani et al. estimated a lower incidence of VN of 3.5 per 100.000 based on population epidemiological survey by questionnaire in Japan (5, 6). In specialized dizziness units, VN accounts for 3–10% of diagnoses among referred subjects (2, 4, 7). In a British general practice study, VN was reported to be the second most common diagnosis after BPPV among subjects suffering from dizziness. According to Literature, too much variability seems to exist in the epidemiological data. The age of onset ranges usually from 30 to 60 years but existing data reported very different mean ages [61.4 (3), 52.3 (4), 44 in male patients and 46 in female patients (5, 6); some studies reported a female predominance (3, 5) while others reported no gender difference (4–6)]. Recurrence rates are ranging from 2 to 11% (8–11) with pathological sequelae such as persistent or chronic disequilibrium, benign paroxysmal positional vertigo (BPPV) (10), and chronic anxiety (11). The present study aims to estimate the incidence of VN in three different districts in Italy, its epidemiological features, and the prevalence of comorbidities associated with it.

## 2. Materials and methods

This prospective cohort study was performed over a 2-year period in the areas of Siena (in 2015), Grosseto (in 2015), and Cuneo (in 2016). Given the relatively low populations in these cities, patients with severe vertigo are referred to a single otoneurological unit in their district. Patients were added to the study according to clinical and diagnostic inclusion criteria and followed for up to 1 year from the onset of symptoms. All the patients were seen by an experienced otoneurologist. Most were sent to the units in the acute stage by emergency departments, and some were sent directly to the respective units by general practitioners. All patients underwent a complete otoneurologic examination in the first 48 h from the onset of symptoms [bedside eye movements examination (12), caloric stimulation, video head impulse (VHIT), Romberg and Fukuda test, subjective visual vertical (bucket test)] (13). Bedside eye movements examination was performed with Frenzel goggles and/or videonystagmography. Caloric irrigation was performed with hot, cold, and ice water, and the asymmetry of vestibular function was calculated using the Jongkees formula (12). The VHIT was performed with a video system (vHIT GN Otometrics, Denmark) on the plane of each canal. The normal gain was >0.8 for lateral canals and >0.7 for vertical canals. Vestibular myogenic evoked potentials (VEMPs) were not performed because not available in all centers at the time of the study. Thus, isolated inferior vestibular neuritis was excluded from this study. Since the exact diagnostic criteria for vestibular neuritis were non-standardized at the time of the study and it is still unclear whether vestibular neuritis is a disorder of the end organ (posterior labyrinth) or nerve (5, 14–17), a very comprehensive diagnostic protocol with strict inclusion criteria was adopted. Vestibular neuritis was diagnosed according to the following major criteria: (a) acute vertigo lasting for at least 24 h; (b) dominantly horizontal-rotatory spontaneous nystagmus beating toward the non-affected ear with a torsional component (beating with the pole at the 12-o'clock position directed toward the non-affected ear); (c) pathologic HIT of VOR function toward the affected side and in the planes of the horizontal canal, (d) pathological caloric stimulation (paresis or paralysis) and VHIT (gain < 0.8); (e) postural unsteadiness with Romberg toward the affected ear; (f) normal otoscopic examination and no unilateral hearing loss or tinnitus associated with vertigo; (g) no additional neurologic signs and symptoms; (h) normal brain images: (MRI in all subjects and CT scan when performed in the emergency setting); and (i) no other vestibular or neurological disorders. Patients with concomitant vestibular disorders, such as Ménière's disease, acute stage lateral semicircular canal paroxysmal positional vertigo, postural phobic vertigo, central vestibular disorders or bilateral vestibular hypofunction, and vestibular migraine, were excluded from the study. If HIT was normal at first referral and a stroke or a transient ischemic attack was suspected, neurologic evaluation and MRI or CT scan were performed in an emergency setting. The majority of the patients underwent a cerebral CT scan according to the internal emergency protocol. All patients underwent brain MRI, during the first 2 months from the onset of symptoms, to exclude brainstem and cerebellar lesions. Furthermore, follow-up lasted for 12 months to check for recurrences, the onset of positional vertigo, or chronic dizziness. The diagnostic criteria adopted fit very well the diagnosis of acute unilateral vestibulopathy/VN defined by the Consensus document of the committee for the International Classification of Vestibular Disorders of the Barany Society (18). Epidemiological data were collected and integrated between the three municipalities, similar from a demographic and climatic point of view, and each with a single otoneurologic unit representing the local center of reference for vestibular disorders (Siena and Grosseto, Tuscany, Central Italy; Cuneo, Piemonte, Northern Italy). Large areas with multiple otoneurological referral centers might be a bias in defining the incidence of VN beacause of a higher leakage in data collection. This was overcome by choosing smaller municipalities and districts with a single emergency department and direct access from the emergency department and general practitioners (24/7 hours a day) to the only otoneurological referral center for the area. Age at onset, sex, and affected side have been analyzed. The following comorbidity conditions were considered: hypertension, diabetes, hyperlipidemia, and migraine. Migraine was diagnosed according to the criteria of the International Headache Society (IHS) (19, 20).

### 2.1. Statistical analysis

Data were analyzed through descriptive statistics (frequency, mean, and standard deviation) to assess the variables' characteristics. The crude and standardized incidence rates were calculated for each area concerning the municipality alone and with its surrounding larger district. The standardization of the incidence rate was assessed for age, using the population data (ISTAT) within the same patient's range. A 95% confidence interval was computed for each estimation. The difference in the mean ages among the samples was verified using the Kruskal–Wallis test since violation of normality was detected by the Kolmogorov–Smirnov test. The two-tailed *Z*-test was used to analyze the difference in the prevalence of a patient's comorbidities compared to that of the population. The significance level was set at *p* < 0.05, and the analyses were performed with IBM SPSS Statistics, version 23 (IBM Corp., Armonk, NY, USA).

## 3. Results

**Figure 1:**
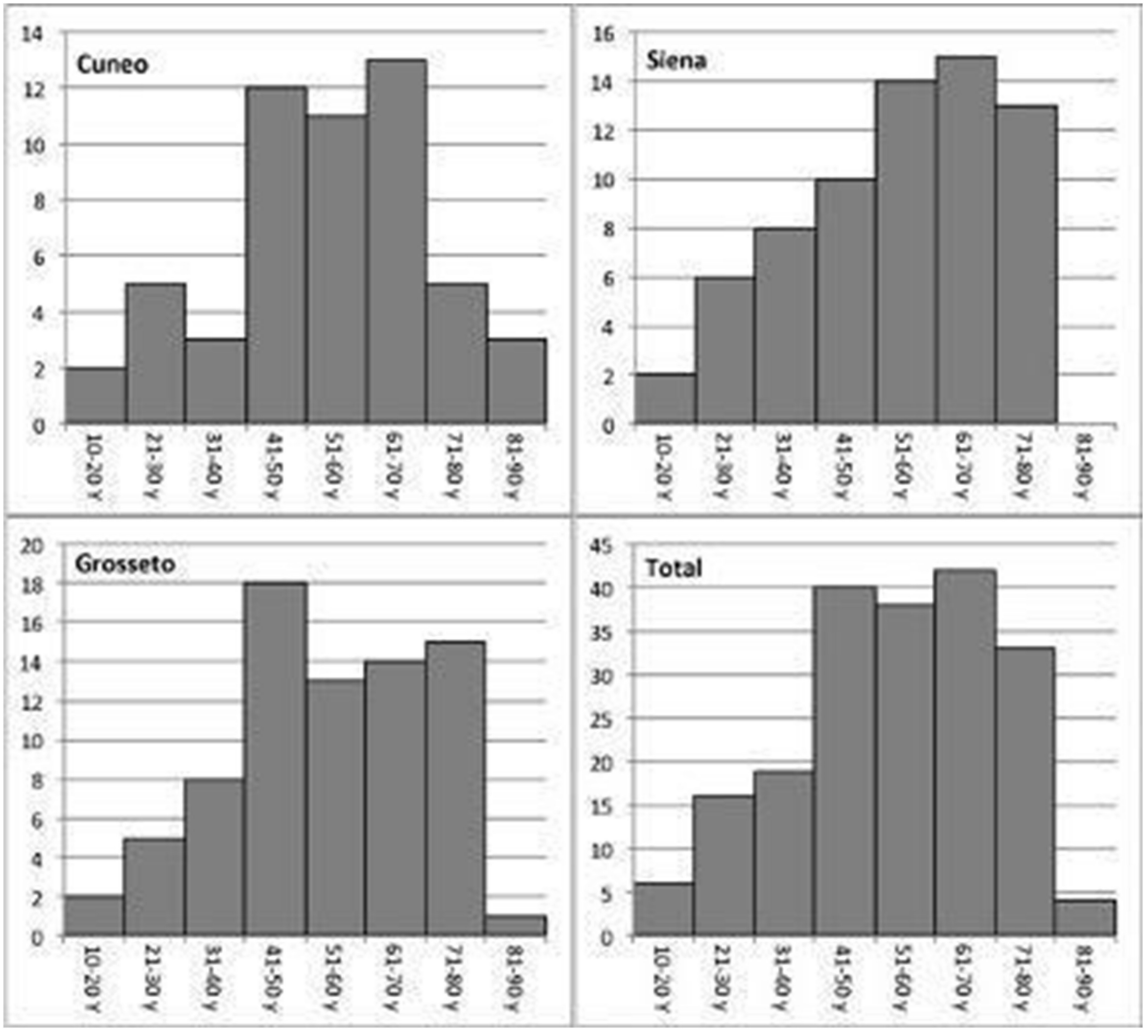
Comparison of age distributions of patients with vestibular neuritis.

**Figure 2:**
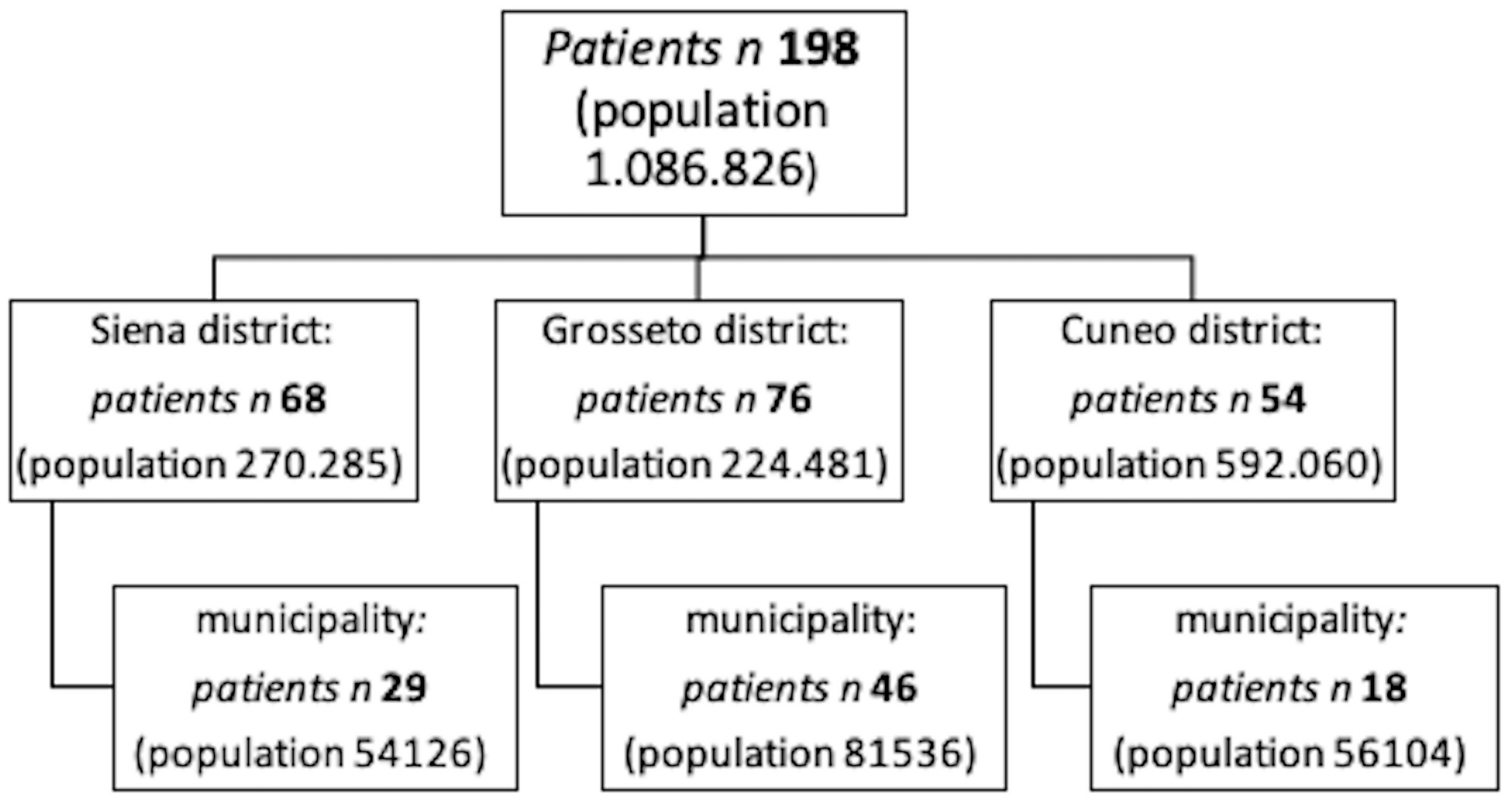
Flow chart of screened patients for each center.

In the study period, a total of 198 patients received the diagnosis of vestibular neuritis according to the criteria. Subjects enrolled met all the inclusion criteria. Of these, 68 were seen in the unit at Siena, 76 in Grosseto, and 54 in Cuneo. As shown in Table 1, 111 patients were male and 87 were female with a total sex ratio of 1.28:1 in favor of the men. The sex ratio showed a clear variability, ranging from 1.06:1 in the Siena district to 1.53:1 in the Grosseto district. The right side was involved in 104 patients and the left side in 94, with a total ratio R/L of 1.1:1, with homogeneous levels of the ratios among the districts. The mean age at onset was 54.13 years (SD: 16.65), ranging from a minimum of 12 years to a maximum of 87 years. Age did not show statistically significant differences among the three districts (K-W = 0.008; *p* < 0.996), but the three samples showed different patterns (Figure 1). The incidence of the VN onset reached its maximum between the ages of 40 and 70 years. Among the 198 subjects enrolled, 29 of them lived in Siena municipality and 39 in its surrounding district, 46 in Grosseto municipality and 30 in its surrounding district, and 18 in Cuneo municipality and 36 in its surrounding district (Figure 2). The cumulative incidence rate was assessed for the total sample of patients enrolled and each district, separating the estimations for the municipalities considered in the study (Table 2). The total incidence rate of the three municipalities was 48.497 (95% CI: 48.395–48.598), and its standardized value was 53.564 (95% CI: 53.463–53.666). The municipality of Grosseto showed the highest incidence rate (56.417; standardized: 62.070), very close to the incidence rate of Siena (53.579; standardized: 59.616), while the lowest value was found for the municipality of Cuneo with an incidence rate of 32.083 (standardized: 35.386). Considering all the patients living in the districts, the incidence rates decreased in magnitude. The total incidence rate was 18.218 (95% CI: 18.164–18.272) and its standardized value was 20.185 (95% CI: 20.129–20.241). The highest rate was again detected in the district of Grosseto and was 33.856 (standardized: 37.111), whereas the district of Cuneo showed a very low incidence rate (9.121; standardized: 10.140), probably because of its larger population resident in the district when compared to the districts of Siena and Grosseto. A group of comorbidities was assessed in a subset of patients, those living in the municipalities of Grosseto and Cuneo. The comparison with the population prevalence during the same period revealed that some of these comorbidities showed a significantly different prevalence than the population data (21–24). In the group of patients, diabetes mellitus, with a prevalence of 15.6% (95% CI: 14.6–16.6%; *p* < 0.000), and migraine-possible (17.2%; 95% CI: 16.2–18.2%; *p* < 0.012) were statistically significantly different from the prevalence of these comorbidities in the population (Supplementary Table 1).

**Table 1:** Sex distribution, affected side distribution and their ratios in the three districts and in the whole sample of patients.

	**Patients (*n*)**	**Gender** ^*^		**M/F ratio**	**Affected side** ^**^		**R/L ratio**
		**M**	**F**		**R**	**L**	
Siena	68	35	33	1.06:1	37	31	1.19:1
Grosseto	76	46	30	1.53:1	40	36	1.11:1
Cuneo	54	30	24	1.25:1	27	27	1.00:1
Total	198	111	87	1.28:1	104	94	1.11:1

^*^M, male; F, female.

^**^R, right; L, left.

**Table 2:** Crude incidence rates, standardized incidence rates (by age) and their 95% confidence interval.

	**Patients**	**Inhabitants**	**Crude incidence rate (95% CI)**	**Standardized incidence rate (95% CI)**
Siena (district)	68	270,285	25.159 (25.055–25.262)	27.912 (27.806–28.019)
Siena (municipality)	29	54,126	53.579 (53.397–53.760)	59.616 (59.437–59.794)
Grosseto (district)	76	224,481	33.856 (33.749–33.962)	37.111 (37.003–37.220)
Grosseto (municipality)	46	81,536	56.417 (56.273–56.560)	62.070 (61.930–62.210)
Cuneo (district)	54	592,060	9.121 (9.044–9.197)	10.140 (10.060–10.221)
Cuneo (municipality)	18	56,104	32.083 (31.868–32.299)	35.386 (35.165–35.607)
Total (all patients)	198	1,086,826	18.218 (18.164–18.272)	20.185 (20.129–20.241)
Total (municipalities)	93	191,766	48.497 (48.395–48.598)	53.564 (53.463–53.666)

## 4. Discussion

In our observational epidemiological study, the crude incidence rate of VN varied from 32.083 (Cuneo municipality) to 56.417 (Grosseto municipality), with a mean value of 48.497 in the three municipalities. This value is slightly higher than that reported previously by other authors (2, 4–6). In our opinion, this rate seems very realistic since we used strict diagnostic criteria, and all dizzy patients converge on a unique referral otoneurologic unit per district working 24/24 h. A significant difference was highlighted between patients residing in the cities compared to those residing in the surrounding area. In our opinion, this may be because general practitioners in the country sometimes manage patients with vertigo independently. In our study, the diagnosis of VN was performed, in most patients, with a bedside vestibular examination: spontaneous nystagmus, head impulse test (HIT), head shaking test (HST), and mastoid vibration. The caloric preponderance and VHIT confirmed the diagnosis. The peripheral origin of the disease was also confirmed by the exclusion of central causes by MRI, performed in all patients during the 2-month period from the onset of vertigo, and by a prolonged follow-up. Indeed, no patients with a diagnosis of VN developed symptoms or signs due to central involvement. Only patients with horizontal or horizontal/torsional spontaneous nystagmus were considered. Therefore, patients with inferior VN were not included in this study since this rarer subtype of VN is classically characterized by spontaneous torsional downbeat nystagmus, abnormal HIT for the posterior semicircular canal, abnormal cervical-VEMPs, normal HIT for the anterior and horizontal semicircular canals, and normal caloric test (15–17). According to our results, VN is 10 times less frequent than BPPV (25), that is, every year, there is a new patient with VN for every 10 with BPPV. When comparing the incidence of VN with that of Ménière's disease (MD), it is well-known that, in the general population, reliable prevalence and incidence estimates of MD are difficult to obtain. Many years ago, we conducted a perspective population-based survey and found that, in Siena, definite MD is rare, with an incidence rate of 5.6 per 100,000 inhabitants per year (26). That study was accurate, and we believe that “true” MD is a rare condition and our value is not far from reality. If so, every year, there is a new patient with MD for every 10 patients with VN. Interestingly, the incidence of VN is similar to that of Bell's palsy, another idiopathic disease probably viral in origin, reinforcing the common causal hypothesis. Indeed, the cumulative incidence of Bell's palsy was 53.3/100,000/year (27). The high incidence of VN documented in our study may be explained in part also with the possible overlap with the diagnosis of vestibular migraine, despite that no patients met the diagnostic criteria for definite VM in the 1-year follow-up. The mean age at the onset of VN was 54.13 years, similar to that in Croatian patients. VN is rare but possible in young people: five patients were teenagers and the youngest was 13 years old. In fact, VN mainly affects adults over 40 years of age, with more than 40% of patients between 40 and 70 years. There was no significant difference between these three decades, and the incidence of the disease does not seem to increase with age. A slight prevalence of the male population was detected in all three cities (111 male patients vs. 87 female patients) that is in accordance with a previously demonstrated study by Adamec et al. and Sekitani et al. (4, 6). This is in contrast to the general behavior of vestibular disorders in adults (BPPV, vestibular migraine, and Meniere's disease). Indeed, the prevalence and incidence rates of vestibular disorders are consistently higher in women than in men (2). There was no statistically significant difference in the site of the lesion with a right/left ratio of 1.11:1. Comorbidities, such as hypertension in 26.6% of patients or diabetes in 15.6%, could suggest shared potential risk factors of vascular origin. On the other hand, the lowest rates of VN in older people > 70 years, when cardiovascular disorders are preponderant, seem to limit this hypothesis. Correlations between VN and migraine (14.1% ICHD-3 defined, 17.2% possible) are possible since the two clinical manifestations share common complaints such as visual vertigo and/or anxiety or phobic vertigo. The scarcity of epidemiological data on vestibular neuritis is however remarkable (2, 5–7). Furthermore, most experienced neurotologic centers all over the world do not have direct access to data from emergency departments making the incidence of VN difficult to estimate and evaluate. The most recent study on VN incidence (4) only evaluated patients over 20 years of age and the population in that study only included one center from the four emergency departments in the area. Finally, it is well-known that it is more difficult for severely impaired dizzy patients to reach the emergency department or neurotological unit promptly in large cities. These limitations may have had a bearing on the lower incidence with respect to our results. There are other limitations to our study. First of all, the sample size. Although the estimations were sufficiently accurate, this does not guarantee that the results can be generalized to the Italian population as a whole, since data were only collected in three small territories in the northwestern and central regions of Italy. Another limitation is related to the analysis of the comorbidities; we assessed them as the difference in prevalence compared to that of the population, but we did not investigate how was the burden of the comorbidities in VN pathogenesis. Finally, since VEMPs were not available in all centers, we excluded the diagnosis of isolated inferior VN from the study. Despite the rarity of isolated inferior VN, this limitation may have lowered the incidence of VN in the present study (16).

## 5. Conclusions

The total VN incidence rate was 18.218/100.000/year and its standardized value was 20.185/100.000/year. The observation of restricted and homogeneous population areas to collect demographic data and a more accurate information from emergency centers and general practioners might lead to a more definite epidemiological observation survey, limiting the effects of diagnostic errors or incorrect pharmacological treatments. Usually, compensation occurs in the days after the acute onset of VN, especially if the patient is rapidly mobilized, so a prompt diagnosis should be made to avoid delays or late/partial recovery. A long-term follow-up of VN patients is however recommended in order to highlight spontaneous compensation/adaptation phenomena to modulate the best vestibular rehabilitation options and to monitor patients for possible recurrences.

## Data availability statement

The raw data supporting the conclusions of this article will be made available by the authors, without undue reservation.

## Ethics statement

Ethical review and approval was not required for the study on human participants in accordance with the local legislation and institutional requirements. Written informed consent for participation was not required for this study in accordance with the national legislation and the institutional requirements.

## Author contributions

MM, CF, and DN contributed to conception and design of the study. SP, SB, SA, and FV organized the database. FF, RG, and GG performed the statistical analysis. GC wrote the first draft of the manuscript. IB, FV, GC, and LS wrote sections of the manuscript. All authors contributed to manuscript revision, read, and approved the submitted version.

## References

[1] Strupp M Magnusson M. Acute unilateral vestibulopathy. Neurol Clin. (2015) 33:669. 10.1016/j.ncl.2015.04.012 26231279

[2] Neuhauser HK. The epidemiology of dizziness and vertigo. In:JM Furman T Lempert, editors, Handbook of Clinical Neurology. Volume 137, Chapter 5. Amsterdam: Elsevier (2016). p. 67–82. 10.1016/B978-0-444-63437-5.00005-4 27638063

[3] Guilemany JM Martínez P Prades E Sañudo I De España R Cuchi A. Clinical and epidemiological study of vertigo at an outpatient clinic. Acta Otolaryngol. (2004) 124:49–52. 10.1080/00016480310002122 14977078

[4] Adamec I Krbot Skoric M HandŽić J Habek M. Incidence, seasonality and comorbidity in vestibular neuritis. Neurol Sci. (2015) 36:91–5. 10.1007/s10072-014-1912-4 25085434

[5] Strupp M Brandt T. Vestibular neurites. Semin Neurol. (2009) 29:509–19. 10.1055/s-0029-1241040 19834862

[6] Sekitani T Imate Y Noguchi T Inokuma T. Vestibular neuronitis: Epidemiological survey by questionnaire in Japan. Acta Otolaryngol Suppl. (1993) 503:9–12. 10.3109/00016489309128061 8470507

[7] Neuhauser H Leopold M von Brevern M Arnold G Lempert T. The interrelations of migraine, vertigo, and migrainous vertigo. Neurology. (2001) 56:436–44. 10.1212/WNL.56.4.436 11222783

[8] Huppert D Strupp M Theil D Glaser M Brandt T. Low recurrence rate of vestibular neuritis: A long-term follow-up. Neurology. (2006) 67:1870–1. 10.1212/01.wnl.0000244473.84246.76 17130428

[9] Kim YH Kim K-S Kim KJ Choi H Choi J-S Hwang IK. Recurrence of vertigo in patients with vestibular neuritis. Acta Otolaryngol. (2011) 131:1172–7. 10.3109/00016489.2011.593551 21728751

[10] Mandalà M Santoro GP Awrey J Nuti D. Vestibular neuritis: Recurrence and incidence of secondary benign paroxysmal positional vertigo. Acta Otolaryngol. (2010) 130:565–7. 10.3109/00016480903311278 19883173

[11] Bronstein A Dieterich M. Long-term clinical outcome in vestibular neuritis. Curr Opin Neurol. (2019) 32:174–80. 10.1097/WCO.0000000000000652 30566414

[12] Mandalà M Nuti D Broman AT Zee DS. Effectiveness of careful bedside examination in assessment, diagnosis, and prognosis of vestibular neuritis. Arch Otolaryngol Head Neck Surg. (2008) 134:164–9. 10.1001/archoto.2007.35 18283159

[13] Zwergal A Rettinger N Frenzel C Dieterich M Brandt T Strupp M. Bucket of static vestibular function. Neurology. (2009) 72:1689–92. 10.1212/WNL.0b013e3181a55ecf 19433743

[14] Büki B Hanschek M Jünger H. Vestibular neuritis: Involvement and long-term recovery of individual semicircular canals. Auris Nasus Larynx. (2017) 44:288–93. 10.1016/j.anl.2016.07.020 27545414

[15] Taylor RL McGarvie LA Reid N Young AS Halmagyi GM Welgampola MS. Vestibular neuritis affects both superior and inferior vestibular nerves. Neurology. (2016) 87:1704–12. 10.1212/WNL.0000000000003223 27694256

[16] Kim JS Kim HJ. Inferior vestibular neuritis. J Neurol. (2012) 259:1553–60. 10.1007/s00415-011-6375-4 22215238

[17] Lee JY Park JS Kim MB. Clinical characteristics of acute vestibular neuritis according to involvement site. Otol Neurotol. (2019) 40:797–805. 10.1097/MAO.0000000000002226 30964776

[18] Strupp M Bisdorff A Furman J Hornibrook J Jahn K Maire R. Acute unilateral vestibulopathy/vestibular neuritis: Diagnostic criteria. J Vestib Res. (2022) 32:389–406. 10.3233/VES-220201 35723133 PMC9661346

[19] Headache Classification Committee of the International Headache Society. The international classification of headache disorders, 3rd edition (beta version). Cephalalgia. (2013) 33:629–808. 10.1177/0333102413485658 23771276

[20] Olesen J. ICHD-3 beta is published. Use it immediately. Cephalalgia. (2013) 33:627–8. 10.1177/0333102413487610 23771275

[21] Tocci G Nati G Cricelli C Parretti D Lapi F Ferrucci A. Prevalence and control of hypertension in the general practice in Italy: Updated analysis of a large database. J Hum Hypertens. (2017) 31:258–62. 10.1038/jhh.2016.71 27629243

[22] ISTAT. Comunicato stampa IL diabete in Italia. Roma: Istituto nazionale di statistica. (2017). Available online at: https://www.istat.it/it/archivio/202600 (accessed July 20, 2017).

[23] Il portale dell'epidemiologia per la sanità pubblica,. Aspetti epidemiologici. (2002). Roma: Istituto superiore di sanità. Available online at: https://www.epicentro.iss.it/colesterolo/epidemiologia

[24] Sarchielli P Granella F Prudenzano MP Pini LA Guidetti V Bono G. Italian guidelines for primary headaches: 2012 revised version. J Headache Pain. (2012) 13:31–70. 10.1007/s10194-012-0437-6 22581120 PMC3350623

[25] von Brevern M Radtke A Lezius F Feldmann M Ziese T Lempert T. Epidemiology of benign paroxysmal positional vertigo: A population based study. J Neurol Neurosurg Psychiatry. (2007) 2007:710–5. 10.1136/jnnp.2006.100420 17135456 PMC2117684

[26] Nuti D Biagini C Passali D. Incidence and prevalence of Menière's disease in Tuscany. In: Menière's Disease: Perspectives in the ‘90s. Proceedings of the 3rd International Symposium on Menière's Disease. Rome (1993). p. 47–9.

[27] Monini S Lazzarino AI Iacolucci C Buffoni A Barbara M. Epidemiology of Bell's palsy in an Italian Health District: Incidence and case-control study. Acta Otorhinolaryngol Ital. (2010) 30:198. 21253285 PMC3008145

