# Supplementary material for: The incidence of vestibular neuritis in Italy
Source: Front Neurol. 2023 May 18;14:1177621. doi: 10.3389/fneur.2023.1177621 (PMC10232831; doi:10.3389/fneur.2023.1177621)
Supplement: Supplementary file 1 [file Table_1.docx]

Supplementary Table 1. Comparison between patient comorbidities and prevalence in the population.

|  | Patients (n) | Population | Statistic* | p |
| --- | --- | --- | --- | --- |
|  |  |  |  |  |
| Hypertension | 26.6% (17) | 25.9% | Z=0.212 | 0.416 |
| Diabetes mellitus | 15.6% (10) | 5.3% | Z=6.488 | **0,000** |
| Hyperlipidaemia | 9.4% (6) | 11.9% | Z=-1.087 | 0.862 |
| Migraine - ICH defined | 14.1% (9) | 12.0% | Z=0.892 | 0.186 |
| Migraine - possible | 17.2% (11) |  | Z=2.247 | **0.012** |
